# Detection and Quantification of House Crickets (*Acheta domesticus*) in the Gut of Yellow Mealworm (*Tenebrio molitor*) Larvae Fed Diets Containing Cricket Flour: A Comparison of qPCR and ddPCR Sensitivity

**DOI:** 10.3390/insects16080776

**Published:** 2025-07-28

**Authors:** Pavel Vejl, Agáta Čermáková, Martina Melounová, Daniela Čílová, Kamila Zdeňková, Eliška Čermáková, Jakub Vašek

**Affiliations:** 1 Department of Genetics and Breeding, Faculty of Agrobiology Food and Natural Resources, Czech University of Life Sciences Prague, Kamýcká 129, 165 00 Prague, Czech Republic; cermakovaagata@af.czu.cz (A.Č.); melounova@af.czu.cz (M.M.); cilova@af.czu.cz (D.Č.); vasek@af.czu.cz (J.V.); 2 Department of Biochemistry and Microbiology, University of Chemistry and Technology Prague, Technická 5, 166 28 Prague, Czech Republic; kamila.zdenkova@vscht.cz (K.Z.); or eliska.cermakova@carc.cz (E.Č.); 3 Department of Food Science, Czech Agrifood Research Center, Drnovská 507/73, 161 00 Prague, Czech Republic

**Keywords:** edible insects, yellow mealworm (*Tenebrio molitor*), house cricket (*Acheta domesticus*), food authentication, gut content analysis, qPCR, ddPCR

## Abstract

Due to their nutritional value and sustainability, edible insect-based foods are gaining popularity in Europe. Their use is regulated by EU legislation, which defines authorised species and sets labelling requirements. Molecular tools are being developed to authenticate such products. In this study, yellow mealworm (*Tenebrio molitor*) larvae authorised for human consumption were fed wheat flour-based diets containing varying proportions of house cricket (*Acheta domesticus*) flour for 21 days. This was followed by a 48 h starvation period to assess the persistence of insect DNA in the digestive tract. Two novel, species-specific, single-copy markers were designed: *ampd* gene for the *Acheta domesticus* and *MyD88* gene for the *Tenebrio molitor*. These were applied using qPCR and ddPCR. Both methods successfully detected cricket DNA in the guts of starved larvae. Linear regression analysis revealed a strong, statistically significant correlation between the proportion of *Acheta domesticus* flour in the diet and the normalised relative quantity of DNA. ddPCR proved to be more sensitive than qPCR, particularly in the detection of low DNA levels. These results suggest that the presence of DNA from undeclared insect species in edible insects may be indicative of their diet rather than contamination or adulteration. This highlights the importance of contextual interpretation in food authenticity testing.

## Simple Summary

In recent years, insects have been recognised as a promising food source thanks to their high nutritional value and low environmental impact. In Europe, only certain species of insect are approved for use in food and feed, and products containing them must be clearly labelled. To help ensure correct labelling, scientists are developing genetic methods to identify the species of insects used in food and feed products. In our study, we focused on yellow mealworm larvae (*Tenebrio molitor*), which are commonly farmed and approved for human consumption. These larvae are usually fed a plant-based diet, but some producers are experimenting with feeding them insect-based flours to enhance their growth and nutritional quality. Therefore, we fed *Tenebrio molitor* larvae diets containing various proportions of house cricket (*Acheta domesticus*) flour and then tested for traces of this feed in their bodies, even after starving them for 48 h. We used two molecular methods (qPCR and ddPCR) to detect insect DNA in the larvae. Both methods were successful, with ddPCR proving to be more sensitive. Our results demonstrate that the presence of another insect’s DNA in mealworms reflects their diet, rather than contamination or fraud, which is important for food labelling and traceability.

## 1. Introduction

As the global demand for sustainable protein sources increases, edible insects are gaining increasing attention as an alternative to traditional livestock. Their high nutritional value, smaller environmental footprint and efficient feed conversion rates make them a promising option for food and feed production. According to Van Huis [1], insect farming offers numerous advantages, including high feed conversion efficiency into animal protein, the potential to utilise organic by-products as food and feed, low greenhouse gas and ammonia emissions, minimal water consumption and a reduced risk of zoonotic disease transmission compared to conventional livestock production. These factors make insects a promising, sustainable alternative to traditional sources of animal protein. However, there are also studies that highlight the potential risks associated with entomophagy. These primarily concern allergenicity and the presence of biological and chemical contaminants [2]. Widespread adoption of entomophagy is hindered by cultural perceptions of insects, as well as concerns about the potential negative effects of insect microbiota on human health [3].

Since 1 January 2018, Regulation (EU) 2015/2283 on novel foods has been in force across the European Union. This regulation clearly defines insects and insect-based products as novel foods. Under its transitional provisions, whole or ground insect bodies from the following species are authorised for sale as food: yellow mealworm larvae (*Tenebrio molitor*), lesser mealworm larvae (*Alphitobius diaperinus*), house cricket adults (*Acheta domesticus*) and migratory locust adults (*Locusta migratoria*).

Larvae of *T. molitor* are among the most widely produced edible insect species in Europe. *T. molitor* is recognised for its nutritional value [4,5], digestibility [6] and bioactive compounds such as antimicrobial peptides and chitin [7]. It is a rich source of stable proteins regardless of diet [8], as well as essential amino acids, beneficial fatty acids, minerals and vitamins. These properties make it a high-energy food suitable for human nutrition [9].

Following their classification as ‘livestock’ under Article 3(6) of Regulation (EC) No 1069/2009 (Regulation (EU) 2017/893), insects were authorised for farming to produce processed animal protein for aquaculture in 2017. Consequently, they can only be reared using feed materials permitted for conventional farm animals, primarily cereal-based substrates. Currently, processed whole insects or insect proteins are authorised for use solely as feed for farmed fish, poultry and pigs, as well as pets and other exotic animals (e.g., reptiles, birds, insectivores or primates). This authorisation covers eight insect species: the black soldier fly (*Hermetia illucens*), the house fly (*Musca domestica*), the yellow mealworm (*T. molitor*), the lesser mealworm (*A. diaperinus*), the house cricket (*A. domesticus*), the banded cricket (*Gryllodes sigillatus*), the Jamaican field cricket (*Gryllus assimilis*) and the silkworm (*Bombyx mori*) [10]. Incorporating insects into the diets of livestock, particularly poultry and pigs, has shown promising effects in terms of enhancing growth and health [11,12]. *T. molitor* [1] and *A. domesticus* represent promising alternatives to conventional protein sources such as soybean meal [13,14].

Some producers of edible insects are considering using other insects as a protein source, primarily due to the potential sustainability and efficiency benefits in terms of feed production. Insects are a highly nutritious and readily available protein source that could reduce our dependence on environmentally demanding conventional animal-based feeds, such as fish meal. Furthermore, using insects as feed could help to close nutrient cycles within insect farming systems, thereby enhancing the sustainability of the production process as a whole. However, current European Union legislation prohibits feeding edible insects other insect species, primarily to prevent the transmission of pathogens and ensure food chain safety. Currently, fish meal is the only authorised source of animal protein in feed for edible insects. This is explicitly outlined in the Transmissible Spongiform Encephalopathy (TSE) Regulation (EU) No 999/2001 and the Animal By-Products (ABP) Regulation (EC) No 1069/2009, which prohibit the use of processed animal proteins, including insects, in feed for farmed insects intended for human consumption. These regulations are designed to ensure traceability, prevent cross-species disease transmission, and protect animal and consumer health within the EU. Additionally, Regulation (EU) 2023/58 requires a mandatory 24 h feed withdrawal period prior to harvesting for the various insect products already approved under Regulation (EU) 2015/2283. This starvation period enables the insects to empty their digestive tracts, thereby improving the hygienic quality of the final product.

In their natural habitat, the *T. molitor* is commonly found under bark, rocks, decaying wood or leaves. There, they consume various types of organic matter, including decaying plant material, fungi and animal remains. They also feed on animal matter, such as meat scraps, dead insects and feathers [15]. Research has shown that mealworm larvae exhibit increased cannibalistic behaviour when they have a lack of water [16] or food [17]. Asendorf et al. [18] provided evidence that *A. diaperinus* exhibits omnivorous feeding behaviour by purposely feeding *A. diaperinus* larvae a mixture of wheat flour and lyophilised pork and chicken meat.

Although typically considered pests in grain storage, mealworms can consume various agricultural by-products and convert them into valuable feed and food [19,20]. They thrive on diets containing both plant and animal matter, although the optimal protein content for growth is around 20% [19]. Commercially, they are usually fed a diet of cereal bran or flour supplemented with fruit and vegetables for hydration, as well as protein sources such as soybean meal, skimmed milk powder and yeast [21]. Recent studies have supported the use of insect flour in insect diets. Morales-Ramos et al. [22], for example, suggest that insect flour is preferable to vertebrate meal for insect production. Meanwhile, Akiyama et al. [23] demonstrated that replacing fish meal with flour from the same species of cricket did not affect the growth of two-spotted crickets (*Gryllus bimaculatus*).

Accurate identification of insect species in food and feed products is essential, as misidentification can pose allergenic risks or lead to consumption of non-approved species [24]. Regulatory frameworks in the European Union and elsewhere require precise labelling and species traceability to ensure food safety and compliance with legal standards [25].

Molecular methods are an effective tool for detecting and identifying insect species in food products, including authorised edible species and potential adulterants. The most commonly used gene for barcoding edible insects is the mitochondrial cytochrome c oxidase subunit I (*COI*) gene. Using *COI* gene polymorphisms, species such as *T. molitor*, darkling beetle (*Zophobas atratus*), *A. diaperinus*, greater wax moth (*Galleria mellonella), B. mori*, *L. migratoria*, desert locust (*Schistocerca gregaria*), *G. bimaculatus*, *A. domesticus* and *H. illucens* have been successfully identified [26,27,28,29,30,31]. The mitochondrial *cytb* gene has been used to identify *G. sigillatus* and *A. domesticus* [32,33], while the mitochondrial *nad4L* gene has been used to detect *T. molitor* [28]. Another commonly used mitochondrial marker is the *16S rRNA* gene [34].

The use of nuclear genes is less frequent. The cadherin gene (*cad*) has been used to distinguish between *T. molitor*, *A. diaperinus* and silkworms [35,36,37]. Other nuclear markers include the wingless gene (*wg*) and the *18S rRNA* gene [35,38].

The most common methods of identifying edible insect species are endpoint PCR [30], quantitative PCR (qPCR) with TaqMan probes [28] and high-resolution melting (HRM) analysis [39]. Another PCR method, droplet digital PCR (ddPCR) shows great promise for identifying and quantifying edible insect DNA. According to McNair et al. [40], ddPCR is considered to be more sensitive, reliable and robust technique than qPCR, especially for detecting small numbers of target DNA molecules. Zhao et al. [41] recommend ddPCR for food authentication and for quantifying the level of food adulteration. However, its direct application to the detection of adulteration in insect-based food products has not yet been reported. Nevertheless, relevant entomological studies do exist. For instance, Zink et al. [42] compared the detection of two *Helicoverpa* moth species using qPCR and ddPCR, confirming the ddPCR method’s superior sensitivity and specificity. Furthermore, Zink et al. [43] developed a duplex ddPCR assay that can detect two bark beetle species simultaneously in bulk trap samples. Another approach to identifying and quantifying edible insect species involves next-generation sequencing (NGS) technologies [44]. Haynes et al. [45] provide an overview of various NGS platforms—such as Illumina, ThermoFisher Scientific, Pacific Biosciences and Oxford Nanopore—that are suitable for food authentication and the detection of food adulteration. Reference genome sequences have been established for the mealworm [46] and *A. domesticus* [47] using NGS, and the knowledge of these whole-genome sequences is essential for the subsequent design of species-specific markers. Hillinger et al. [34] used the MiSeq^®^ and iSeq^®^ 100 platforms from Illumina to sequence part of the *16S rRNA* mitochondrial gene to identify different species of edible insect.

Molecular analyses also investigate the trophic relationships between insects and their food sources. For example, Huang et al. [48] identified prey in the gut of the predatory damsel bug (*Eocanthecona furcellata*) by amplifying a fragment of the *COI* gene using PCR. The presence of molluscan DNA representing carabid beetle prey items was confirmed by amplifying a fragment of the *18S rRNA* gene [49]. Gut content analysis is conducted in insects from both natural and laboratory environments. Asendorf et al. [18], for example, detected pork and chicken meat in the digestive tract of *A. diaperinus* larvae using a combination of multiplex PCR assays, real-time PCR, DNA biochip technology and short- and long-read NGS platforms.

Building on these approaches, our study focused on detecting insect-derived feed DNA in *T. molitor* larvae using advanced molecular techniques. Our aim was to assess the potential of these techniques to trace the dietary history of farmed insects and improve traceability in insect-based food production systems.

The main objectives of this study were as follows: (i) to design species-specific single-copy nuclear gene markers for detecting *A. domesticus* DNA in *T. molitor* larvae, (ii) to compare the sensitivity of ddPCR and qPCR in identifying residual insect DNA post-feeding and (iii) to evaluate the impact of a 24 h starvation period on detection accuracy. We hypothesised that ddPCR would exhibit greater sensitivity than qPCR, and that a positive correlation exists between the dietary proportion of cricket flour and the amount of detectable DNA in larval gut samples. Additionally, we proposed that starving larvae prior to sampling would facilitate detection of residual DNA from insect-based feed. Finally, we aimed to clarify whether the presence of undeclared insect DNA in samples reflects the dietary history of the larvae rather than contamination or adulteration of the feed.

## 2. Materials and Methods

### 2.1. Biological Material and Experimental Design

The experiment was conducted using actively feeding *T. molitor* larvae measuring approximately 10–12 mm in length. The larvae were at a developmental stage characterised by continued growth, moulting and intensive feeding. Larvae were sourced from commercial breeding facilities, where they were originally intended as feed for exotic animals. Experimental diets were formulated by supplementing coarse wheat flour (*Triticum aestivum*) with varying proportions of house cricket (*A. domesticus*) flour intended for human consumption (Lordy, Jablonec nad Nisou, Czech Republic). The resulting diets contained 0%, 25%, 50%, 75% and 100% *A. domesticus* flour.

### 2.2. Rearing Conditions and Experimental Treatments

The larvae were reared in 100 mL glass Erlenmeyer flasks at 27 °C and 63% relative humidity in the dark using a thermostat-controlled incubator Q-Cell (Pol-Lab, Wilkowice, Poland). Each flask contained 5 g of the designed diet and 25 larvae approximately 15 mm in length. The experiment was conducted in two biological replicates. After 21 days, 20 larvae from one replicate were euthanised by immersion in boiling deionised water for three minutes. This treatment group is referred to as ‘non-starved larvae’. Larvae from the second replicate were transferred to clean Erlenmeyer flasks without food and maintained for an additional 48 h at 27 °C. They were then euthanized using the same method and classified as the ‘starved’ group. Following euthanasia, all larvae were rinsed with clean deionised water, dried on filter paper for three hours, and then lyophilised in polypropylene tubes (Christ Alpha, Osterode am Hartz, Germany) for 72 h. The lyophilised samples were homogenised in Minilys homogenizer (Bertin Instruments, Montigny-le-Bretonneux, France).

### 2.3. DNA Extraction

DNA was extracted from 15 mg of lyophilised and homogenised larvae using a NucleoSpin^®^ Tissue Kit (Macherey-Nagel, Düren, Germany) in accordance with the manufacturer’s protocol for animal tissues. To assess the specificity of the newly designed qPCR markers, DNA was also isolated from several other insect species: *Z. atratus*, *A. diaperinus*, *G. bimaculatus*, red-jawed cricket (*Gryllus locorojo*), *G. sigillatus*, *L. migratoria*, *S. gregaria*, giant cave cockroach (*Blaberus giganteus*), discoid cockroach (*Blaberus discoidalis*), Argentine cockroach (*Blaptica dubia*), Turkestan cockroach (*Shelfordella lateralis*), *H. illucens*, common green bottle fly (*Lucilia sericata*), *B. mori* and greater wax moth (*Galleria mellonella*), as well as several vertebrate species: human (*Homo sapiens*), cattle (*Bos taurus*), domestic pig (*Sus domesticus*), chicken (*Gallus gallus*), turkey (*Meleagris gallopavo*), mallard (*Anas platyrhynchos*) and common carp (*Cyprinus carpio*). DNA was also extracted from selected plant species that could potentially serve as feed for *T. molitor*, including wheat (*T. aestivum*), maize (*Zea mays*), soybean (*Glycine max*), potato (*Solanum tuberosum*), carrot (*Daucus carota*) and apple (*Malus domestica*). DNA concentrations were determined using a spectrophotometric method (NanoPhotometer, Implen, Germany).

### 2.4. Primer and Marker Design

Two highly species-specific nuclear single-copy genes were selected to identify and quantify DNA from the *A. domesticus* (*ampd* gene for AMP deaminase) and the *T. molitor* (*MyD88* gene, which encodes the myeloid differentiation primary response protein). For *A. domesticus*, the primer pair Ad_AMPD_F (5′-TGTAAAACGACGGCCAGTTTGATAAGGATGTCCCTGCCCCAG-3′) and Ad_AMPD_R (5′-CAGGAAACAGCTATGACCAGTGGTTCAATACAGTCAAGTTCGCA-3′) was designed to amplify a 141 bp product, based on the NCBI GenBank sequence GQ888288.1. The primer pair Tm_MyD88_F (5′-TGTAAAACGACGGCCAGTATGAAGTTCTGCGTGAAAGACCGC-3′) and Tm_MyD88_R (5′-CAGGAAACAGCTATGACCTCCGAGAGGATGACGATCAAACGG-3′) was designed to amplify a 146 bp product in *T. molitor*, based on the NCBI GenBank sequence NC_091048.1. All primers were designed using Primer3 version 4.1.0 [50]. The underlined sequences represent M13 adapters [51], which were used for the sequencing of PCR amplicons.

### 2.5. Quantitative Polymerase Chain Reaction (qPCR)

qPCR reactions with a total volume of 10 µL were prepared in technical triplicates for each marker. Each reaction mixture contained 5.0 μL of FastStart Essential DNA Green Master (Roche, Basel, Switzerland), 40 ng of genomic DNA and 0.25 μM of each primer. Amplification and melting curve analyses were performed in thin-walled, hard-shell PCR plates (Bio-Rad, Hercules, CA, USA) using a CFX Connect Real-Time PCR System (Bio-Rad, Hercules, CA, USA), under the following conditions: an initial denaturation step at 95 °C for 600 s, followed by 40 cycles of denaturation at 95 °C for 20 s, annealing at 69 °C for 20 s and extension at 72 °C for 20 s, with a ramp rate of 5 °C/s between each step. For the melting curve analysis, the temperature increased from 65.0 to 95.0 °C in increments of 0.5 °C/s.

The normalised relative quantification (*RQ_norm_*) of the DNA content of *A. domesticus* in the digestive tracts of *T. molitor* larvae was performed using the ΔΔCt method described by Livak and Schmittgen [52]. The amplification of target DNA (*ampd* gene from the *A. domesticus*) was normalised against reference DNA (*MyD88* from *T. molitor*) and compared with a calibrator sample (*T. molitor* larvae that were fed a diet containing 100% *A. domesticus* flour and were not starved). The calculation was carried out as follows:

$${RQ}_{norm}\;=\;2^{-\Delta \Delta Ct}$$

$$\Delta Ct={\;Ct}_{target}-{Ct}_{reference}$$

$$\Delta \Delta Ct={\Delta Ct}_{sample}-{\Delta Ct}_{calibrator}$$

where

- *Ct_target_* is the threshold cycle (*Ct*) of the target gene *ampd*.
- *Ct_reference_* is the *Ct* of the reference gene *MyD88*.
- Δ*Ct_sample_* is the Δ*Ct* value of the test sample.
- Δ*Ct_calibrator_* is the Δ*Ct* value of the calibrator sample.

This method only applies if both primer pairs have the same amplification efficiency. Based on minimal differences in the slope values (less than 0.1), it has been confirmed that the efficiencies can be considered identical, as reported by Livak and Schmittgen [52].

### 2.6. Droplet Digital PCR (ddPCR)

The ddPCR mixture, prepared separately for each marker, consisted of a total volume of 20 µL and consisted of 10 µL of 2× ddPCR EvaGreen Supermix (Bio-Rad, Hercules, CA, USA), 40 ng of genomic DNA and 40 µM of each primer. The ddPCR mix (20 μL) and Droplet Generation Oil for EvaGreen^®^ (70 μL) were combined using the QX200^TM^ Droplet Generator (Bio-Rad, Hercules, CA, USA). The resulting 40 µL of droplets were immediately transferred to a 96-well plate (Bio-Rad, Hercules, CA, USA), sealed with aluminium foil and placed in the deep-well block of a T100^TM^ Thermal Cycler (Bio-Rad, Hercules, CA, USA). The two-step PCR amplification protocol consisted of an initial denaturation step at 95 °C for 300 s, followed by 40 cycles of denaturation at 95 °C for 30 s and annealing/extension at 65 °C for 40 s. This was followed by a final incubation step of 4 °C for 300 s, 90 °C for 300 s and 10 °C for 300 s, with a ramp rate of 2.5 °C/s between each step.

After amplification, the plate was incubated at 20 °C for 15 min before being transferred to the QX200^TM^ Droplet Reader (Bio-Rad, Hercules, CA, USA). Positive and negative droplets were counted using QuantaSoft software version 1.7.4.0917 (Bio-Rad, Hercules, CA, USA).

Absolute quantification of the marker genes was performed using a ddPCR assay. The concentrations (copies/20 μL) were determined separately for the *MyD88* gene (*T. molitor*) and the *ampd* gene (*A. domesticus*). As both target genes are single-copy nuclear genes, relative quantification (*RQ*) was calculated using the following formula:

$$RQ\;=\;\frac{C_{AMPD}}{C_{MyD88}}$$

where

- *C_AMPD_* is the absolute concentration (copies/20 μL) of the *A. domesticus ampd* gene.
- *C_MyD88_* is the absolute concentration (copies/20 μL) of the *T. molitor MyD88* gene.

To enable comparison between samples, the resulting *RQ* ratio was normalised using a calibrator sample. This was defined as *T. molitor* larvae that were fed a 100% *A. domesticus* diet and were not starved. The final normalised relative quantification (*RQ_norm_*) was calculated as follows:

$${RQ}_{norm}\;=\;\frac{{RQ}_{sample}}{{RQ}_{calibrator}}\;=\;\frac{C_{AMPD\;(sample)}/C_{MyD88\;(sample)}}{C_{AMPD\;(calibrator)}/C_{MyD88\;(calibrator)}}$$

### 2.7. Calculation of the Starvation Effect

The starvation effect (*S_Eff_*), which is defined as the relative reduction in the amount of *A. domesticus* DNA (*ampd* gene) in the guts of *T. molitor* due to starvation, was quantified using the following formula:

$$S_{Eff}\;\left(\%\right)\;=\;\left(1-\;\frac{{RQ}_{norm\;\left(starved\right)}}{{RQ}_{norm\;\left(non-starved\right)}}\right)\;\times 100$$

where

- *RQ_norm (starved)_* is normalised relative quantification of *ampd* gene from *A. domesticus* detected in starved *T. molitor* larvae.
- *RQ_norm (non-starved)_* is normalised relative quantification of *ampd* gene from *A. domesticus* detected in non-starved *T. molitor* larvae.

### 2.8. Sequencing of PCR Products

Following electrophoresis, amplicons of both genes (*MyD88* and *ampd*) were excised from a 3% agarose gel and purified using a MiniElute PCR Purification Kit (Qiagen, Germany). Sequencing was performed using the BigDye Terminator v3.1 Kit (Life Technologies, Carlsbad, CA, USA) on an ABI 310 DNA Analyzer (Life Technologies, Carlsbad, CA, USA). The M13_F (5′-TGTAAAACGACGGCCAGT-3′) and M13_R (5′-CAGGAAACAGCTATGACC-3′) sequencing primers were used, as described by Messing [51]. Consensus sequences were assembled using BioEdit software version 7.0.5.3 [53]. To verify the specificity of the designed markers, sequence alignments were conducted using the international nucleotide database GenBank (NCBI) [54].

### 2.9. Statistical Analysis

A linear regression and correlation analysis was conducted to examine the relationship between the normalised relative concentration (*RQ_norm_*) of *A. domesticus* DNA in the digestive tract of *T. molitor* larvae. Experiments in which the larvae were fed only wheat flour, without *A. domesticus* flour, were considered as negative controls. In these cases, the *A. domesticus*-specific marker (*ampd* gene) did not amplify. Only variants with positive detection of *A. domesticus* in the digestive tract of *T. molitor* larvae (with 25%, 50%, 75% or 100% *A. domesticus* flour in the feed) were used for regression and correlation analyses. The strength of the association was assessed using the coefficient of determination (R^2^). The analysis was performed using R software version 4.4.3 [55].

### 2.10. Ethical Approval

All experimental procedures were conducted in accordance with Czech legislation (Section 29 of Act No. 246/1992 Coll. on the Protection of Animals Against Cruelty, as amended by Act No. 77/2004 Coll.). We hereby declare that the handling of animals in this study complied with the relevant European and international guidelines on animal welfare. Specifically, it complied with Directive 2010/63/EU on the protection of animals used for scientific purposes, as well as the guidelines and recommendations of the Federation of European Laboratory Animal Science Associations (FELASA). Moreover, according to Czech legislation, insects are not classified as laboratory animals; therefore, ethical approval was not required for the experiments conducted in this study.

## 3. Results

### 3.1. Larval Development on Different Feeding Substrates

Standard larval development was observed across all feeding substrate variants during the 21-day rearing period. The larvae actively consumed food, moulted and grew. No larvae died of natural causes during this time. In the final days of the rearing period, a small number of larvae exhibited reduced mobility as they prepared for their final moult and pupation. These larvae were excluded from subsequent analyses. Visually, no differences in larval size or colour were observed in relation to the feeding substrate variant.

### 3.2. Extracted DNA and Its Parameters

DNA suitable for PCR analysis was extracted from all experimental variants, including the control samples (other animal and plant species), using a NucleoSpin^®^ Tissue Kit (Macherey-Nagel, Düren, Germany). Minimal fragmentation and the absence of RNA were confirmed by gel electrophoresis on a 1% agarose gel. The yield of genomic DNA across the experimental variants ranged from 7.3 to 9.5 ng per 1 mg of lyophilised material. Absorbance ratios (A_260_/A_280_) ranged from 1.87 to 1.93, and A_260_/A_230_ values ranged from 1.96 to 2.28.

### 3.3. Species Specificity of the Newly Designed Markers

**Figure 1:**
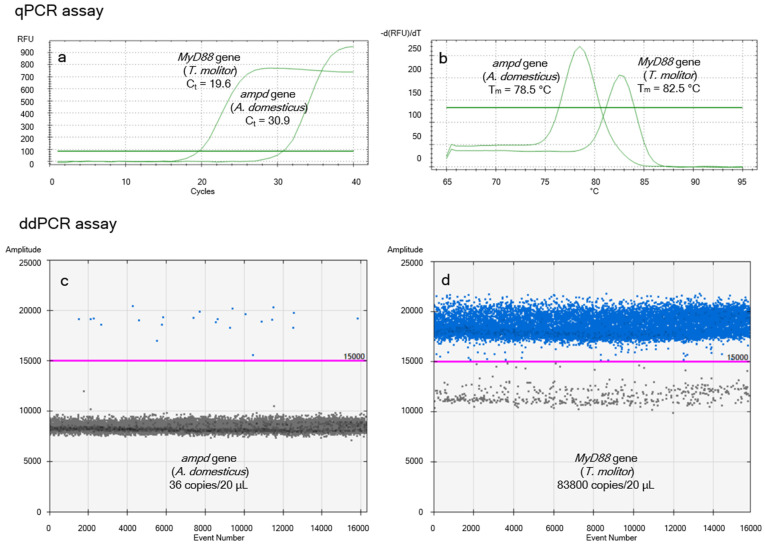
Representative results of *A. domesticus* quantification in the digestive tract of non-starved *T. molitor* larvae fed a diet containing 50% *A. domesticus* flour. (**a**) Amplification curves from the qPCR assay. (**b**) Melting temperature analysis from the qPCR assay. (**c**) Copy number of the *ampd* gene (*A. domesticus*) determined by ddPCR. (**d**) Copy number of the *MyD88* gene (*T. molitor*) determined by ddPCR.

The species specificity of the amplicons was verified using the NCBI BLASTN v.2.17.0+ tool [56]. The results of the in silico analysis predicted the species specificity of the *ampd* gene for the *A. domesticus* and the *MyD88* gene for *T. molitor*. This was experimentally confirmed. Using both amplification platforms (qPCR and ddPCR), amplification occurred exclusively in *A. domesticus* and *T. molitor*, respectively. No amplification was observed in other reference animal or plant species. Figure 1 shows representative amplification profiles for *T. molitor* larvae reared on a diet containing 50% *A. domesticus* flour without starvation.

The specificity of the designed markers was also confirmed by sequencing. The partial sequences of the *A. domesticus ampd* gene detected in the gut contents of *T. molitor* larvae were deposited in the NCBI GenBank database under the accession numbers PV719348–PV19351 for larvae without starvation, and under PV719352–PV19355 for larvae subjected to starvation. Similarly, the partial sequences of the *T. molitor MyD88* gene (from larvae fed with *A. domesticus* flour) were submitted to the NCBI GenBank database under the accession numbers PV719347 and PV719356–PV719359 for larvae without starvation, and under PV719360–PV719364 for larvae subjected to starvation. The sequence of the *A. domesticus ampd* gene from the *A. domesticus* flour used in the preparation of the dietary variants was deposited in the NCBI GenBank database under the accession number PV719365. All newly obtained sequences showed 100% identity with the reference sequences GQ888288.1 (*ampd*) and NC_091048.1 (*MyD88*), respectively.

The Supplementary Materials include Table S1, which presents the raw data (Ct_AMPD_, Ct_MyD88_, *RQ_norm_* and SD) from the qPCR analyses. Table S2 provides the raw data from the ddPCR analyses (*C_AMPD_*, *C_MyD88_*, *RQ_norm_* and SD). Figure S1a,b shows the amplification curves for the non-starved and starved variants, respectively. Figure S2a,b shows the results of the melting curve analysis for the non-starved and starved variants, respectively. Figures S3 and S4 show the number of positive and negative droplets obtained from the ddPCR analysis of the non-starved and starved variants, respectively.

### 3.4. Regression and Correlation Analysis

**Figure 2:**
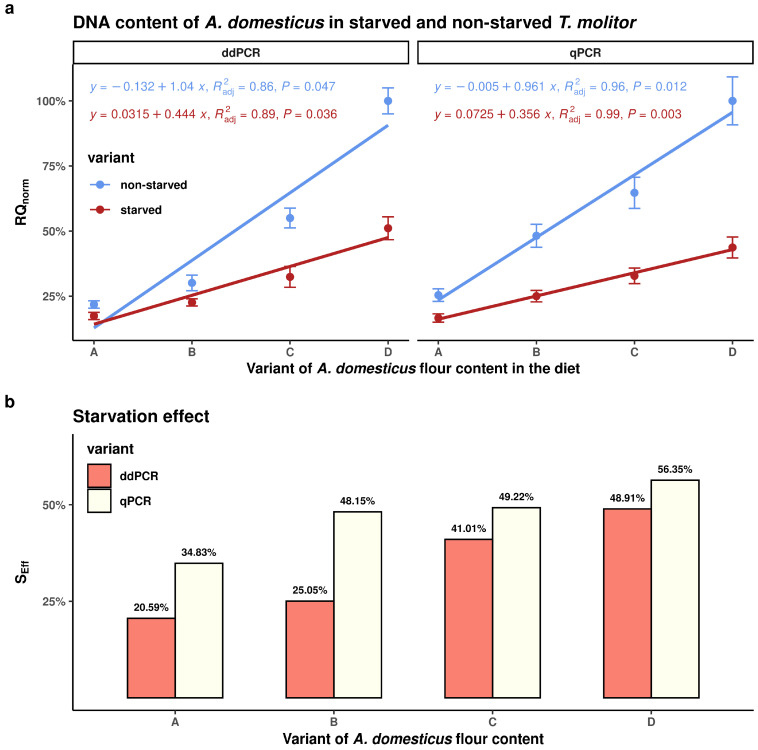
Comparison of ddPCR and qPCR methods via regression and correlation analysis (**a**) and starvation effect (**b**). In both (**a**) and (**b**), groups A, B, C, and D represent different proportions of *A. domesticus* flour in the diet of *T. molitor*: A = 25%, B = 50%, C = 75%, and D = 100%.

As illustrated in Figure 2a, there was a strong positive correlation between the percentage of *A. domesticus* flour in the diet and the relative quantification (*RQ_norm_*) of *A. domesticus* DNA in the digestive tract of *T. molitor* larvae under non-starved and starved conditions. While both ddPCR and qPCR demonstrated a strong correlation, the qPCR method showed a closer dependence, as evidenced by a higher coefficient of determination (R^2^). This indicates that qPCR provides a better model fit. However, the error bars show that there is greater variability among replicate measurements in the qPCR results than in those obtained using ddPCR.

### 3.5. Starvation Effect

Figure 2b shows that the magnitude of the starvation effect (*S_Eff_*) was directly proportional to the percentage of *A. domesticus* flour in the feeding substrate. The same figure also clearly shows that the relative starvation effect was consistently higher across all dietary variants when assessed using qPCR compared to ddPCR. This discrepancy is due to the ddPCR method being more sensitive than qPCR.

## 4. Discussion

### 4.1. T. molitor and A. domesticus as Subjects of Our Research

This study focused on the larval stage of *T. molitor,* one of the insect species officially approved for human consumption under EU Regulation (EU) 2015/2283. Within the EU, *T. molitor* larvae are among the most commonly consumed insect species and are typically served whole, either fried or roasted, and seasoned with salt or a variety of spices. *T. molitor* was selected primarily due to the ease and reliability of its species-level identification, as reported by Park et al. [57]. Due to its holometabolous development (i.e., complete metamorphosis), selecting larvae at a consistent developmental stage based on body length was relatively straightforward. We assumed that larvae measuring around 15 mm would have ample time to grow during the 21-day period while remaining in the larval stage and avoiding pupation. However, determining the exact instar was not feasible as the total number of larval moults is reported to range between 15 and 17 [57]. Nevertheless, the presence of exuviae served as a marker of larval growth and, therefore, of food intake, including consumption of diets supplemented with *A. domesticus* flour. Another reason for choosing *T. molitor* larvae were also ideal due to their morphology: a smooth, glossy cuticle with minimal setae and no wings or wing pads, which reduces the risk of substrate contamination. *T. molitor* is also considered an ideal model organism due to its low husbandry requirements. The environmental conditions employed—27 °C and 63% relative humidity—were consistent with those used in both experimental setups and commercial rearing, as described by Mirzaeva et al. [58]. The larvae’s inability to climb the smooth, conical glass walls of the rearing vessels eliminated the need for covers, minimising the risk of cross-contamination between experimental groups and inhibiting fungal growth within the feeding media. The omnivorous feeding behaviour of *T. molitor* larvae also supported their selection. Their nutritional flexibility has been recognised since Cotton [15] documented wild populations feeding on various organic materials, including meat residues, dead insects and feathers. In this study, the base feeding substrate consisted of coarse wheat flour. Plant-based meals, bran and plant by-products such as these are commonly found in standard insect diets, as described by Jankauskienė et al. [59]. The mealworm’s omnivorous diet is evidenced by its ability to metabolise various substrates, including sweet potato and turnip leaves [60], herbs such as oregano, thyme, sage and rosemary [61], chestnut shells [62] and even polystyrene [63]. Cannibalism, which has been observed under suboptimal rearing conditions [16], further supports the species’ capacity to utilise animal-based materials as a nutritional source. The limited number of studies on feeding *T. molitor* with animal protein-enriched substrates is likely a consequence of EU regulations prohibiting the use of animal-derived waste in insect feed. Nevertheless, Asendorf et al. [18] conducted a comparable experiment with a different species, the *A. diaperinus*, in which the larvae were fed lyophilised pork and chicken for seven days and successfully accepted this animal-based diet. Although an experiment involving *A. diaperinus* could have been conducted, this species was deliberately excluded due to its minimal utilisation as an edible insect in the Czech Republic. Between 2023 and 2025, only one commercially available product containing *A. diaperinus*—an insect-based protein blend—was present on the Czech market.

*A. domesticus*, which we used as a supplement to the larval feeding substrate in our study, is approved under European Union legislation for human consumption and as livestock feed. For the preparation of various experimental feed mixtures, we selected commercially available *A. domesticus* flour intended for human nutrition based on several considerations. We assumed that, as the flour met the quality standards required for human consumption, it would also be suitable for larval development and not negatively affect the growth of *T. molitor*. Although multiple producers of *A. domesticus* flour operate within the Czech Republic, the nutritional profiles of these products are highly comparable. The cricket flour contained 100% *A. domesticus* and provided the following nutritional values per 100 g: 70 g protein, 20 g fat (including 5.2 g saturated fat), 0.5 g carbohydrates, 9.5 g fibre and salt 0.8 g. The randomly selected product was dark brown in colour, had a fine, powdery texture and a neutral taste. It also had a pleasant, mildly caramel-like aroma. Despite being processed and sold in the Czech Republic, the product label indicated that the crickets originated from Thailand. None of the producers provided information on specific production processes, such as the method used to kill and dry the crickets, homogenisation or potential defatting steps. Although *A. domesticus* flour was the main animal-based feed used in the study, *A. diaperinus* flour (Entoway, Brno, Czech Republic) was also considered as an alternative product. However, this product was excluded from the study due to its intense aroma, reminiscent of cinnamon and cocoa, which could have influenced the feeding behaviour of the larvae. We hypothesised that such strong flavourings might cause *T. molitor* larvae to reject the feed. Another advantage of the *A. domesticus* flour used in our study was its fine, powdery consistency, which enabled highly homogeneous mixtures to be prepared with wheat flour. This uniform distribution was crucial in preventing selective feeding, as the larvae were less able to distinguish or avoid specific components (plant vs. animal) within the feed. A comparable approach was reported by Asendorf et al. [18], who fed *A. diaperinus* larvae powdered, lyophilised pork and chicken meat. Unlike our study, their experimental design involved feeding the larvae an exclusively animal-based diet, with pure wheat flour used only as a negative control.

### 4.2. Nuclear or Mitochondrial Genes as Tools for Authenticating Insect-Based Foods and Quantifying Potential Adulteration

Ratnasingham and Hebert [64] report that the most commonly utilised genes for insect barcoding in the Barcode of Life Data System (BOLD) database are selected mitochondrial genes. For *T. molitor*, the database currently contains 485 publicly available records, most of which are based on fragments of the mitochondrial *COI* gene, typically ranging from approximately 560 to 670 bp. Similarly, for *A. domesticus*, there are 148 records, with *COI* sequences of a similar length predominating. As our study focused on detecting insect DNA in processed food products, where extensive DNA fragmentation is expected due to diverse processing technologies, the amplicon lengths commonly used in barcoding were considered too long. However, *COI* and other mitochondrial gene fragments ranging from 100 to 250 bp are often employed for detecting edible insects in food matrices [26,27,28,29,30,31,32,33]. As these analyses aim to confirm species authenticity or detect adulteration, the benefits of using mitochondrial markers are clear. The number of mitochondria per somatic cell varies with tissue type and developmental stage, typically ranging from hundreds to thousands [65]. The high copy number and circular structure of mtDNA increase the likelihood of successful PCR amplification, even in highly degraded food DNA samples.

However, when the quantitative analysis of individual species in complex mixtures is required, nuclear single-copy genes offer a more precise alternative. These genes are also used for insect food authentication [35,36,37,38], but they are less frequently applied due to the limited number of gene copies per cell—typically two—which can present a challenge when working with fragmented DNA. The primary objectives of our study were to detect *A. domesticus* DNA in the digestive tract of *T. molitor* larvae and to develop species-specific nuclear markers for both species. This decision was based on previously reported issues with species specificity in some published nuclear markers [35]. In silico analyses predicted the amplification of a fragment of the *ampd* gene in *A. domesticus* and of the *MyD88* gene in *T. molitor*. These predictions were confirmed experimentally. Amplicon lengths and melting curve analyses related to the qPCR assay (see Figures S1a,b and S2a,b in the Supplementary Materials) supported its specificity. This was further confirmed by sequencing of the amplicons deposited in the NCBI GenBank database. Figures S3 and S4 in the Supplementary Materials demonstrate that the primer pairs designed for the ddPCR assay produced only a minimal raining effect. The positive and negative droplets were clearly separated, forming two distinct populations with well-defined fluorescence amplitudes.

As detailed in the DNA Extraction section, species specificity was further validated against 15 insect species commonly reared for human and animal consumption, 7 vertebrate species and 6 plant species. The primer pair Ad_AMPD_F and Ad_AMPD_R exclusively amplified a 141 bp fragment in *A. domesticus*, even in samples taken from the digestive tracts of *T. molitor* larvae that had been fed diets containing *A. domesticus* flour. In contrast, the primer pair Tm_MyD88_F and Tm_MyD88_R specifically amplified a 146 bp fragment of the *MyD88* gene in *T. molitor*. We propose that the nuclear markers developed in this study are more suitable for quantifying dietary insect DNA, as they reduce the variability associated with mitochondrial markers, which exhibit high copy number variation across different cell types.

### 4.3. Comparative Sensitivity of qPCR and ddPCR

Hou et al. [66] summarise the advantages of ddPCR over qPCR. These include absolute quantification without the need for standard calibration curves, increased sensitivity and precision in detecting low copy numbers of target DNA, greater resistance to PCR inhibitors and improved reproducibility. These features make ddPCR particularly well-suited to the detection of food adulterants [67,68,69]. We propose applying ddPCR to authenticate specific insect-based food products or quantify potential adulterants. However, to date, no scientific studies targeting this application specifically have been published. Consequently, the results of our pilot experiments represent a novel contribution to the field. One likely reason for the absence of such studies is the relatively high cost of ddPCR analysis. For instance, Hamaguchi et al. [70] state that analysing 96 samples using ddPCR costs approximately three times more than qPCR ($470 vs. $156). Furthermore, many food quality control laboratories, especially those affiliated with government regulatory bodies, may not have the advanced equipment required for ddPCR analysis.

In our pilot study, we compared the performance of ddPCR with qPCR, which is more commonly employed in the genetic analysis of edible insects and is more affordable and technically less demanding. While previous studies using qPCR, such as those by [27,28,36,37], have centred on identifying declared insect species using TaqMan probes, they have not addressed the quantitative detection of adulterants.

Nevertheless, there are studies focusing on the entomological applications of molecular diagnostics. For instance, Zink et al. [42] compared the sensitivity of qPCR and ddPCR in detecting two species of the *Helicoverpa* genus. Their study demonstrated the significantly higher sensitivity of ddPCR, enabling the detection of a single individual’s DNA against a background of DNA from 999 individuals of another species. Interestingly, both their studies targeted the Internal Transcribed Spacer 1 (ITS1) region of the nuclear genome, which is part of the 18S-5.8S-28S rRNA operon and varies in copy number across insect genomes [71]. In contrast, our analysis targeted two strictly single-copy genes: *ampd* (in *A. domesticus*) and *MyD88* (in *T. molitor*). Therefore, the quantity of target DNA in the Zink et al. [42] study was inherently higher due to the multi-copy nature of ITS1. Nevertheless, our results confirmed the superior sensitivity of ddPCR over qPCR in detecting and quantifying *A. domesticus* DNA in the digestive tracts of *T. molitor* larvae.

In our comparison, we performed both qPCR and ddPCR analyses simultaneously on identical sample sets, using different amplification kits: FastStart Essential DNA Green Master (Roche, Switzerland) was used for qPCR and ddPCR EvaGreen Supermix (Bio-Rad, Hercules, CA, USA) was used for ddPCR. For both methods, optimising the template DNA concentration was essential to ensure reproducibility. Optimal amplification for qPCR was achieved using 40 ng of template DNA per 10 μL reaction, yielding Ct values of 29.11–32.74 for *ampd* gene and 19.02–19.95 for *MyD88* gene (see Table S1 in Supplementary Materials). For ddPCR, the optimal template concentration was 40 ng per 20 μL. At this concentration, both genes produced clearly distinguishable populations of positive and negative droplets. The corresponding amplicon concentrations ranged from 11.4 to 139.0 copies/20 μL for *ampd* gene and 37,540 to 101,600 copies/20 μL for *MyD88* gene (see Table S2 in Supplementary Materials). Notably, the template DNA concentration used in ddPCR was half than used in qPCR, highlighting ddPCR’s enhanced sensitivity. Due to gene-specific amplification efficiencies, identical template concentrations could not be used for both methods. Despite these differences, ddPCR yielded lower variability among replicates, consistent with the findings of Hou et al. [66] and as evidenced by the shorter error bars (±2 SD) in Figure 2b and the raw data in Supplementary Materials (Tables S1 and S2).

Our findings are further supported by a recent study by Asendorf et al. [18], the only researchers, to our knowledge, who have investigated the molecular detection of animal-derived dietary components in the gut of *A. diaperinus*. While our study focused on *T. molitor*, a species that primarily consumes plant-based diets, *A. diaperinus* typically consumes a more varied diet that includes animal-derived materials, such as poultry manure [72]. Therefore, feeding *A. diaperinus* chicken meat or pork, as in the Asendorf et al. [18] study, is more aligned with its natural feeding behaviour. However, both studies had the same goal: to compare molecular techniques for identifying dietary components at the species level within insect digestive tracts. Asendorf et al. [18] fed their larvae lyophilised chicken and pork for seven days; our experiment involved feeding *T. molitor* larvae cricket (*A. domesticus*) flour for 21 days, which may have increased the likelihood of detecting target DNA.

Furthermore, in contrast to the study by Asendorf et al. [18], our research showed that both qPCR and ddPCR techniques could detect *A. domesticus* DNA, even when the *A. domesticus* flour represented only a portion of the feed (25%, 50% or 75%). This enabled us to confirm the hypothesis that the concentration of detected cricket DNA in the digestive tract of *T. molitor* positively correlates with the proportion of *A. domesticus* flour in the diet. As with our results, Asendorf et al. [18] concluded that molecular genetic techniques offer high sensitivity and enable the identification of species-specific dietary components in *A. diaperinus*, even when insects are fasted prior to harvest. This period is often used to reduce residual feed and associated gut microbiota [73,74,75]. Our study was the first to introduce the starvation effect (S_Eff_) concept, which is defined as a relative measure of the decline in *A. domesticus* DNA in the digestive tract of *T. molitor*. We found that the SE value positively correlated with the proportion of *A. domesticus* flour in the feed, and that its magnitude depended on the molecular method used. The lower SE observed with ddPCR is likely due to its superior sensitivity compared to qPCR.

When it comes to the practical application of molecular genetic techniques—particularly metabarcoding—in the authentication of edible insects, sensitivity, accuracy and reproducibility are all critical factors. However, the requirements for laboratory equipment and the complexity of subsequent bioinformatic data processing are equally important. Asendorf et al. [18] also highlighted this when they compared conventional PCR (targeting the *COI* gene), a biochip assay (Meat 5.0 LCD Array Kit, Chipron, Berlin, Germany) and NGS via Oxford Nanopore Technologies. Consistent with our findings, they concluded that the species origin of feed components in insect digestive tracts can be reliably determined using even the most cost-effective and technically straightforward PCR methods.

### 4.4. Legislation Versus Edible Insects Fed with Other Insect Species

Current European Union legislation, including Regulations (EU) 2017/893 and (EU) 2015/2283 on novel foods and (EU) 1169/2011 on the provision of food information to consumers, does not permit insects to be used as feed for other insects intended for human consumption. Only specific categories of feed materials, such as fruit, vegetables, cereals or certain plant-based by-products, are authorised for the rearing of edible insects. In this context, the presence of DNA from one insect species (e.g., *A. domesticus*) in the digestive tract of another species intended for human consumption (e.g., *T. molitor*) could raise questions about compliance under existing legislation. However, it is important to emphasise that such findings would not constitute intentional food adulteration or cross-contamination during processing. Rather, they reflect the outcome of controlled feeding practices that are not yet fully addressed within the current regulatory framework.

Looking ahead, future revisions to EU legislation may reflect the growing interest in the principles of the circular bioeconomy, sustainable agriculture and using insects as an alternative source of protein. Provided that appropriate safety assessments are carried out, feeding edible insects to other insect species may be permitted. If this is authorised, the list of approved feed materials and labelling requirements would need to be amended accordingly. In particular, product labelling would need to transparently reflect the identity of the consumed insect species, as well as relevant information about its feeding history—especially if such information impacts food safety or allergenicity. In this regard, the molecular detection of residual DNA from feed sources could be a useful tool for regulatory control and consumer transparency.

## 5. Conclusions

This study involves the development and validation of two original single-copy nuclear genomic markers for the species-specific detection and quantification of two authorised edible insect species within the EU: *T. molitor* and *A. domesticus*. The marker designed for *A. domesticus* targets the *ampd* gene, while the *T. molitor* marker targets the *MyD88* gene. Two methods were successfully applied—qPCR and ddPCR. High sensitivity and reproducibility were demonstrated by both methods, with ddPCR being particularly effective at detecting low DNA concentrations.

This research also represents the first documented attempt to detect insect-derived feed components within the digestive tract of another insect species. A strong positive correlation was observed between the amount of *A. domesticus* DNA detected and the proportion of *A. domesticus* flour in the feed mixture, even after a starvation period in *T. molitor* larvae. These results demonstrate the potential of molecular techniques for authenticating insect-based food products and monitoring dietary inputs throughout the insect farming process.

From a regulatory perspective, current EU legislation prohibits the use of insect proteins as feed for farmed insects because insects are classified as ‘animal proteins’. Consequently, the presence of DNA from a different insect species in an edible insect could be considered non-compliance with legislation, even if it does not constitute intentional adulteration or post-processing contamination.

Looking ahead, the use of insects as feed for other insects is increasingly being discussed as a sustainable, circular approach to protein production. If such a practice were to be permitted in the future under strictly controlled conditions, substantial revisions to EU legislation would be required. These would include clear definitions for the labelling of insect-based foods produced under such feeding regimes to ensure transparency for both producers and consumers. In such a scenario, molecular markers like those developed in this study could play a vital role in traceability and regulatory compliance within insect farming systems.

## Data Availability

Raw data obtained from qPCR and ddPCR analyses are presented in Supplementary Materials. Sequencing data have been deposited in the NCBI GenBank database under the following accession numbers: PV719347–PV19365.

## Supplementary Materials

The following supporting information can be downloaded at: https://www.mdpi.com/article/10.3390/insects16080776/s1, Figure S1: Raw data for the quantification of *A. domesticus* in the gut of *T. molitor* under non-starved conditions by qPCR: (a) amplification curves; (b) melting temperature (Tm) analysis; Figure S2: Raw data for the quantification of *A. domesticus* in the gut of *T. molitor* under starved conditions by qPCR: (a) amplification curves; (b) melting temperature (Tm) analysis; Figure S3: Raw data (positive and negative droplets) from the ddPCR assay for the variant without starved *T. molitor* larvae. Panels A. B. C. and D show diets containing 25%. 50%. 75%. and 100% *A. domesticus* flour. respectively; Figure S4: Raw data (positive and negative droplets) from the ddPCR assay for the variant with starved *T. molitor* larvae. Panels A. B. C. and D show diets containing 25%. 50%. 75%. and 100% *A. domesticus* flour. respectively; Table S1: Raw data for the relative quantification of *A. domesticus* in the digestive tract of *T. molitor* by qPCR assay; Table S2: Raw data for the relative quantification of *A. domesticus* in the digestive tract of *T. molitor* by ddPCR assay.

## Abbreviations

The following abbreviations are used in this manuscript:

16S rRNA	RNA located in the small subunit of mitochondrial ribosomes
18S rRNA	RNA located in the small subunit of eukaryotic ribosomes
A260/A230	Ratio of UV absorbance measured at 260 nm and 230 nm
A260/A280	Ratio of UV absorbance measured at 260 nm and 280 nm
ABP	Animal by-products
*ampd*	Gene encoding adenosine monophosphate deaminase
bp	Base pairs
C	Absolute concentration
*cad*	Gene encoding cadherin protein
*COI*	Gene encoding mitochondrial cytochrome c oxidase subunit I
Ct	Threshold cycle
ddPCR	Droplet digital polymerase chain reaction
DNA	Deoxyribonucleic acid
EC	European Community
FELASA	Federation of European Laboratory Animal Science Associations
HRM	High-resolution melting analysis
ITS1	Internal transcribed spacer 1
M13	Filamentous bacteriophage M13
mtDNA	Mitochondrial deoxyribonucleic acid
*MyD88*	Gene encoding myeloid differentiation primary response protein 88
NCBI	National Center for Biotechnology Information
NGS	Next-generation sequencing
PCR	Polymerase chain reaction
qPCR	Quantitative polymerase chain reaction
R^2^	Coefficient of determination
RFU	Relative fluorescence units
RQ	Relative quantification
SEff	Starvation effect
TSE	Transmissible spongiform encephalopathy
*wg*	Gene encoding wingless protein in insects
