# Supplementary material for: Detection and Quantification of House Crickets (Acheta domesticus) in the Gut of Yellow Mealworm (Tenebrio molitor) Larvae Fed Diets Containing Cricket Flour: A Comparison of qPCR and ddPCR Sensitivity
Source: Insects. 2025 Jul 28;16(8):776. doi: 10.3390/insects16080776 (PMC12386928; doi:10.3390/insects16080776)
Supplement: Supplementary file 1 [file insects-16-00776-s001.zip › Insects-3722291-Supplementary.pdf]

Supplementary Materials

Table S1. Raw data for the relative quantification of *A. domesticus* in the digestive tract of *T. molitor* by qPCR assay

Variant	*A. domesticus* flour content in feed [%]	Ct_{AMPD}	Ct_{MyD88}	RQ_{norm}	SD
non-starved	25	31.37	19.47	0.25437	0.01167
		31.60	19.34		
		31.41	19.43		
non-starved	50	30.90	19.57	0.48187	0.02211
		30.71	19.70		
		30.64	19.62		
non-starved	75	30.32	19.40	0.64692	0.02968
		30.16	19.49		
		30.08	19.57		
non-starved	100	29.14	19.02	1.00000	0.04588
		29.18	19.11		
		29.11	19.09		
starved	25	32.74	19.93	0.16576	0.00761
		32.48	19.95		
		32.45	19.80		
starved	50	31.10	19.12	0.24986	0.01146
		31.20	19.07		
		31.30	19.20		
starved	75	31.58	19.78	0.32850	0.01507
		31.35	19.85		
		31.39	19.66		
starved	100	30.38	19.18	0.43654	0.02003
		30.41	19.13		

30.46 19.15

Table S2. Raw data for the relative quantification of *A. domesticus* in the digestive tract of *T. molitor* by ddPCR assay

**Variant**	***A. domesticus* flour content in feed [%]**	**C_{AMPD} [copies/20 µL]**	**C_{MyD88} [copies/20 µL]**	**RQ_{norm}**	**SD**
non-starved	25	26	91600	0.21849	0.00664
		27	91600		
		28	88000		
non-starved	50	36	83800	0.30115	0.01478
		34	82000		
		33	84400		
non-starved	75	74	100800	0.54951	0.01941
		72	97600		
		78	99800		
non-starved	100	136	98400	1.00000	0.02529
		134	101600		
		139	99200		
starved	25	14.9	62400	0.17407	0.00835
		15.9	63800		
		14.6	64600		
starved	50	11.8	37540	0.22572	0.00716
		12	38240		
		11.4	38300		
starved	75	39	85600	0.32416	0.01962
		37	81300		
		35	83600		
starved	100	48	65400	0.51089	0.02197
		46	65400		
		44	66800	0.21849	0.00664

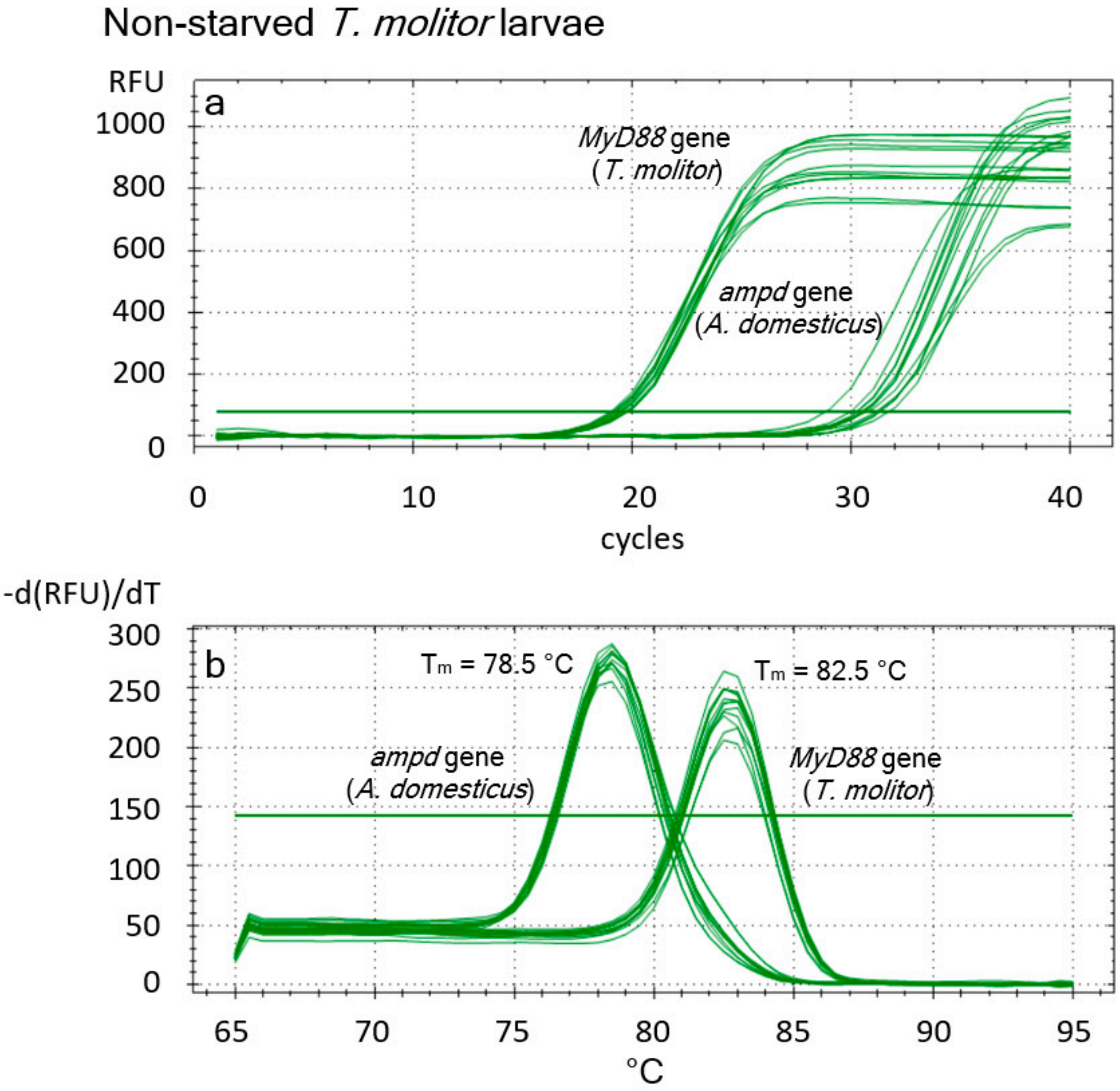

Figure S1. Raw data for the quantification of *A. domesticus* in the gut of *T. molitor* under non-starved conditions by qPCR: (a) amplification curves; (b) melting temperature (Tm) analysis

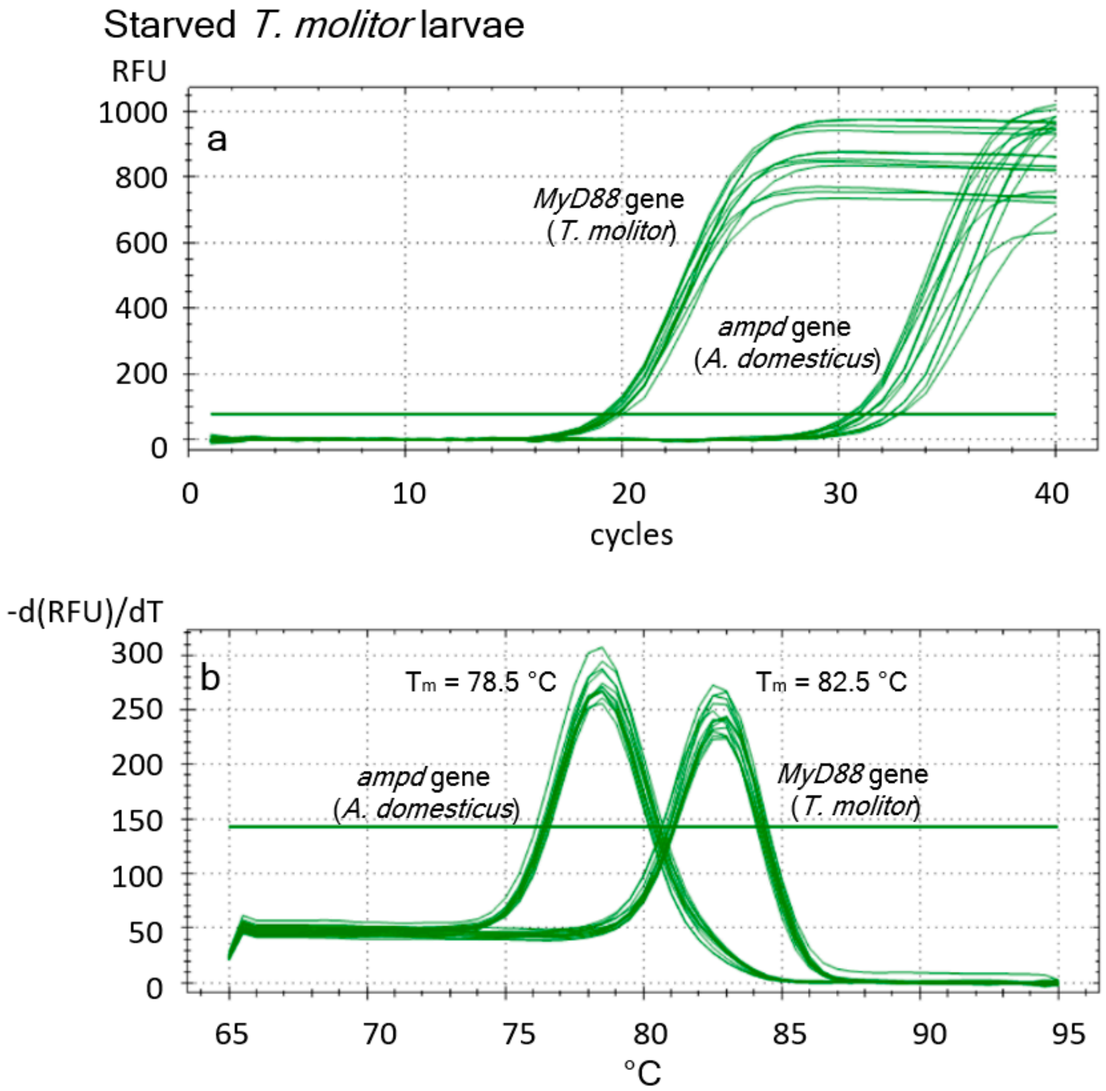

Figure S2. Raw data for the quantification of *A. domesticus* in the gut of *T. molitor* under starved conditions by qPCR: (a) amplification curves; (b) melting temperature (Tm) analysis

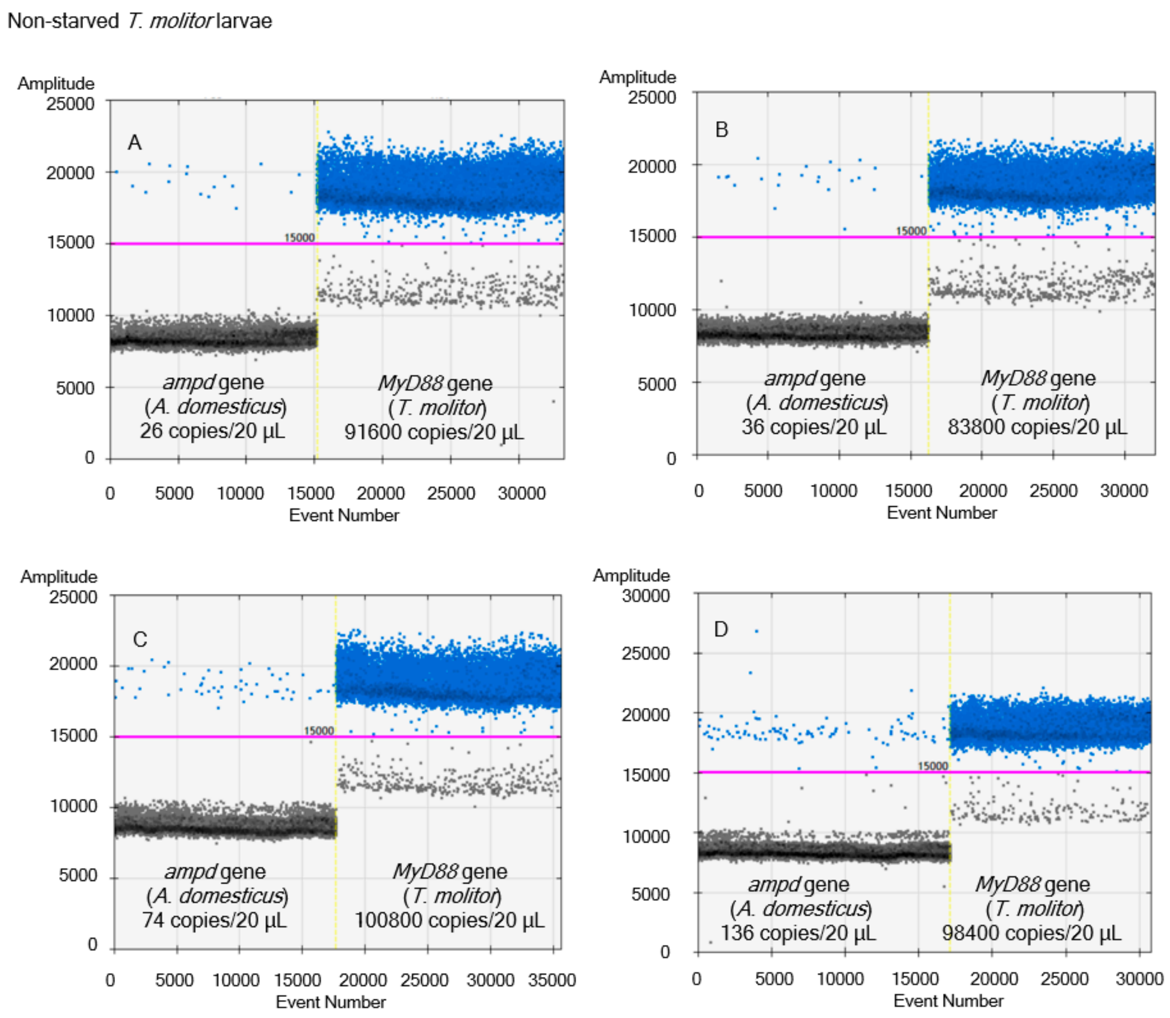

Figure S3. Raw data (positive and negative droplets) from the ddPCR assay for the variant without starved *T. molitor* larvae. Panels A. B. C. and D show diets containing 25%. 50%. 75%. and 100% *A. domesticus* flour. respectively.

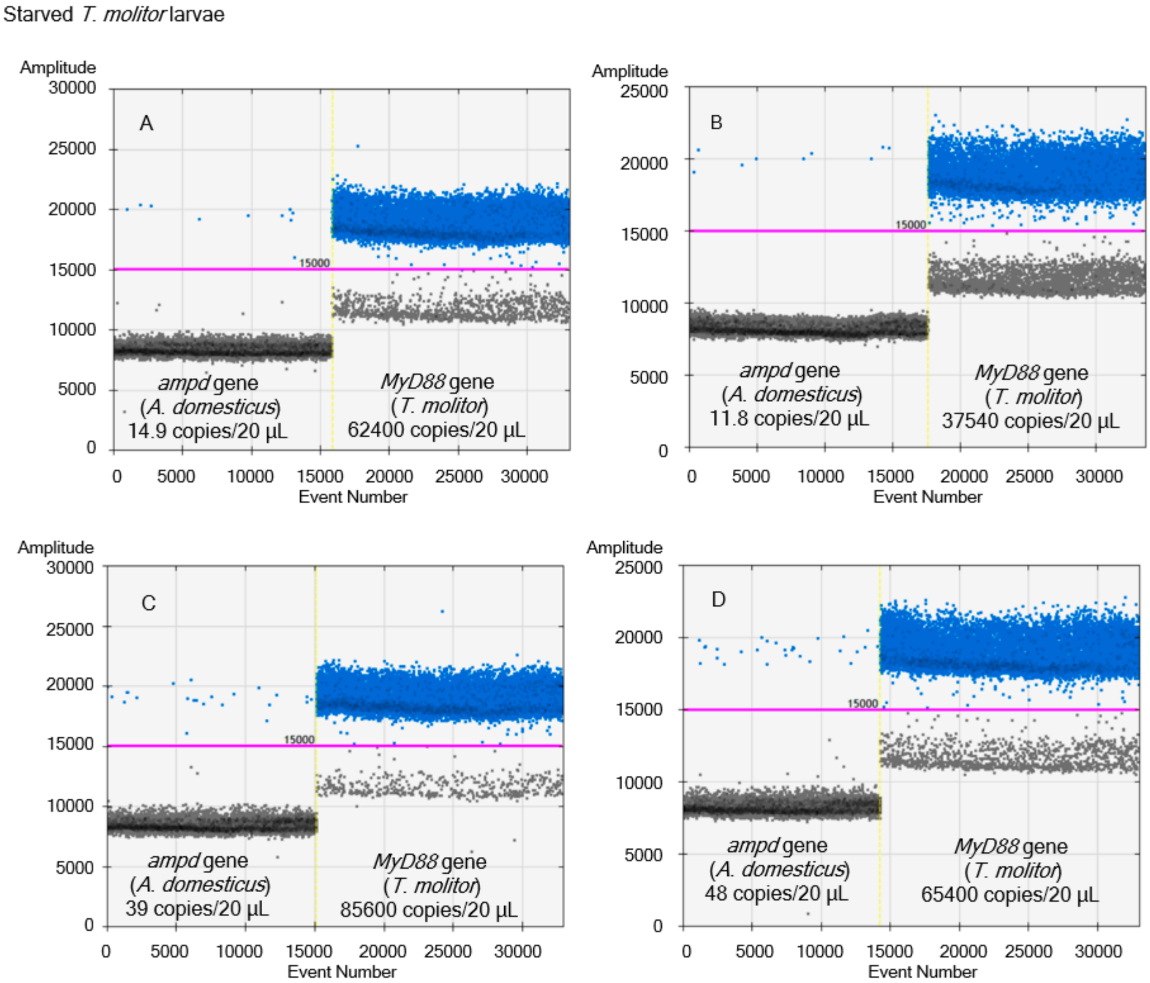

Figure S4. Raw data (positive and negative droplets) from the ddPCR assay for the variant with starved *T. molitor* larvae. Panels A. B. C. and D show diets containing 25%. 50%. 75%. and 100% *A. domesticus* flour. respectively.
