# Supplementary figures and images for: Detection and Quantification of House Crickets (Acheta domesticus) in the Gut of Yellow Mealworm (Tenebrio molitor) Larvae Fed Diets Containing Cricket Flour: A Comparison of qPCR and ddPCR Sensitivity
Source: Insects. 2025 Jul 28;16(8):776. doi: 10.3390/insects16080776 (PMC12386928; doi:10.3390/insects16080776)

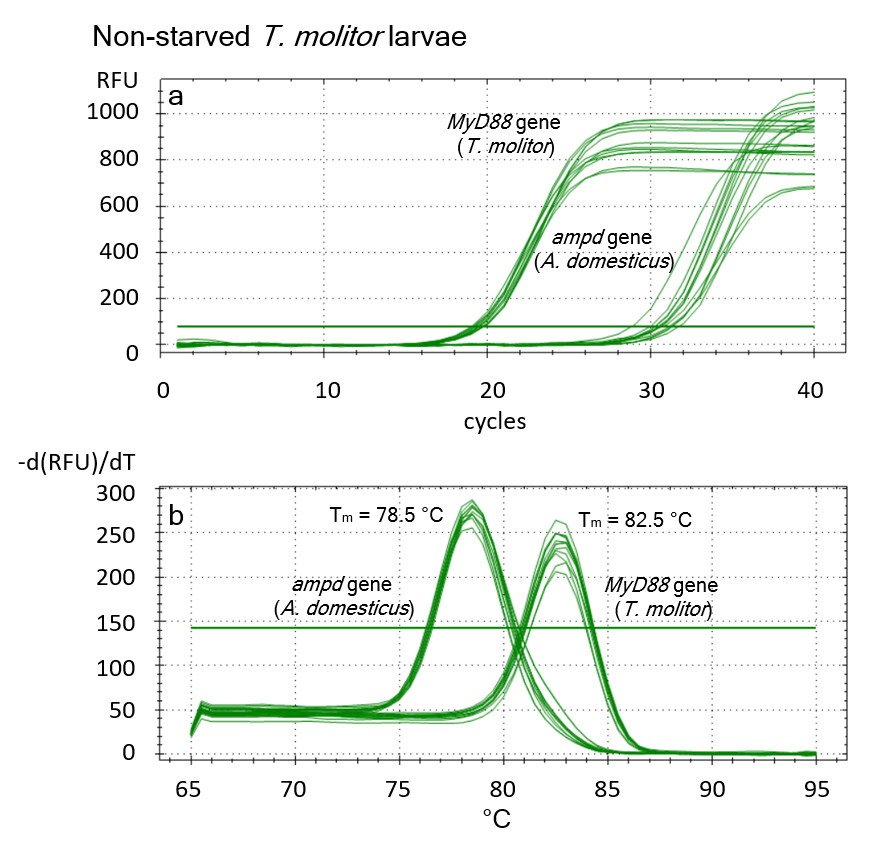

Supplement: Supplementary file 1 [file insects-16-00776-s001.zip › Figure S1.tif]

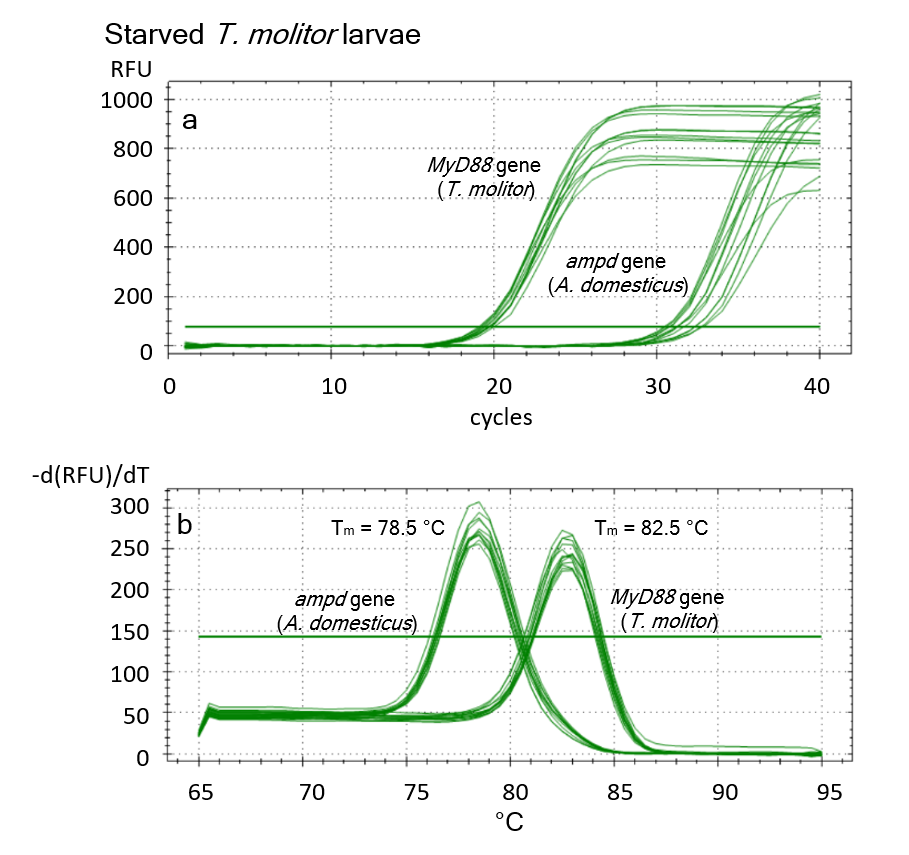

Supplement: Supplementary file 1 [file insects-16-00776-s001.zip › Figure S2.tif]

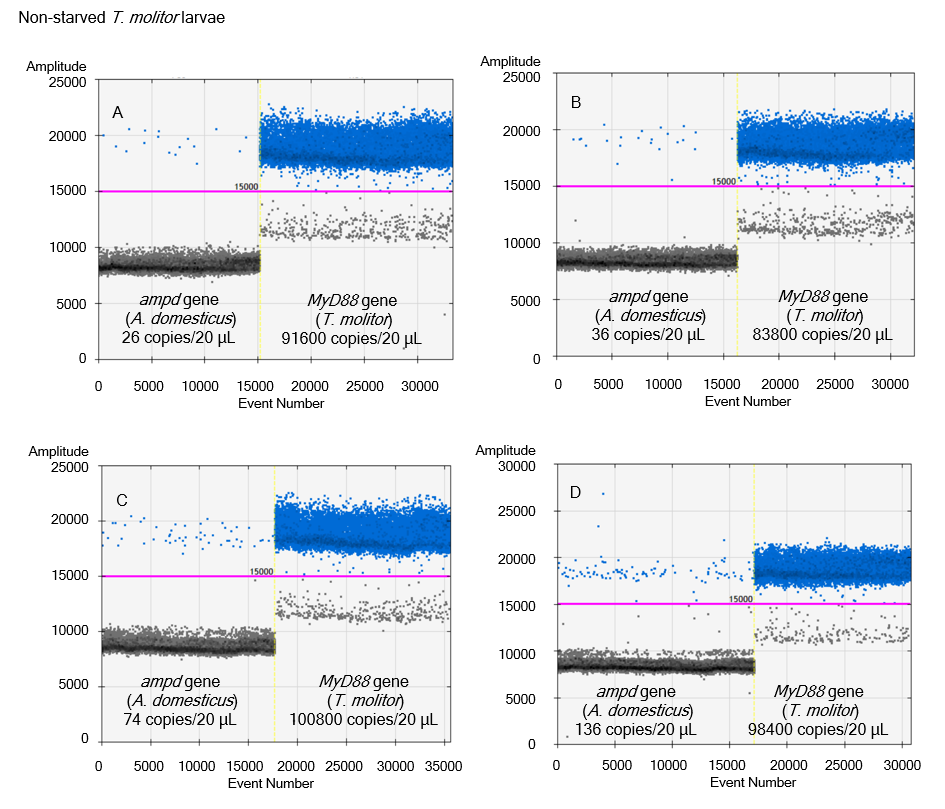

Supplement: Supplementary file 1 [file insects-16-00776-s001.zip › Figure S3.tif]

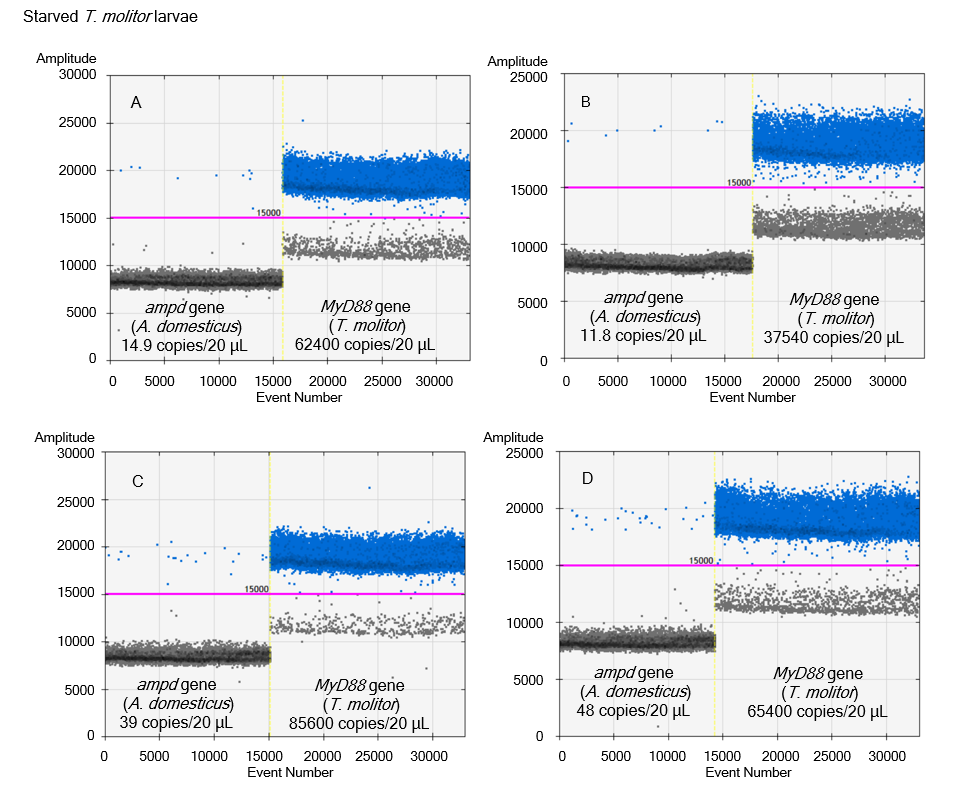

Supplement: Supplementary file 1 [file insects-16-00776-s001.zip › Figure S4.tif]
